# Selective Laser Sintering of PA 2200 for Hip Implant Applications: Finite Element Analysis, Process Optimization, Morphological and Mechanical Characterization

**DOI:** 10.3390/ma14154240

**Published:** 2021-07-29

**Authors:** Răzvan Păcurar, Petru Berce, Anna Petrilak, Ovidiu Nemeş, Cristina Ştefana Miron Borzan, Marta Harničárová, Ancuţa Păcurar

**Affiliations:** 1 Department of Manufacturing Engineering, Faculty of Industrial Engineering, Robotics, Management and Production Management, Technical University of Cluj-Napoca, B-dul Muncii 103–105, 400641 Cluj-Napoca, Romania; petru.berce@tcm.utcluj.ro (P.B.); anna_petrilak@yahoo.com (A.P.); 2 Department of Environmental Engineering and Sustainable Development Entrepreneurship, Faculty of Materials and Environmental Engineering, Technical University of Cluj-Napoca, B-dul Muncii 103–105, 400641 Cluj-Napoca, Romania; ovidiu.nemes@imadd.utcluj.ro; 3 Department of Electrical Engineering, Automation and Informatics, Faculty of Engineering, Slovak University of Agriculture in Nitra, Tr. A. Hlinku 2, 949 76 Nitra, Slovakia; marta.harnicarova@uniag.sk; 4 Department of Mechanical Engineering, Faculty of Technology, Institute of Technology and Business in České Budějovice, Okružní 10, 370 01 České Budějovice, Czech Republic

**Keywords:** selective laser sintering, hip implants, acetabular liner, surface roughness, compression, finite element analysis, contact pressure, paraxylene immersion

## Abstract

Polyamide 12 (PA 22000) is a well-known material and one of the most biocompatible materials tested and used to manufacture customized medical implants by selective laser sintering technology. To optimize the implants, several research activities were considered, starting with the design and manufacture of test samples made of PA 2200 by selective laser sintering (SLS) technology, with different processing parameters and part orientations. The obtained samples were subjected to compression tests and later to SEM analyses of the fractured zones, in which we determined the microstructural properties of the analyzed samples. Finally, an evaluation of the surface roughness of the material and the possibility of improving the surface roughness of the realized parts using finite element analysis to determine the optimum contact pressure between the component made of PA 2200 by SLS and the component made of TiAl6V4 by SLM was performed.

## 1. Introduction

Selective laser sintering (SLS) is an additive manufacturing (AM) method that is widely used to produce prostheses for human bone replacement for skull and hip joint implants [1,2,3,4,5,6]. SLS technology allows obtaining all the required components of a total hip replacement with the same equipment [7]. This advantage makes SLS technology one of the most competitive AM technologies used in the medical domain. The use of a biocompatible material for SLS is one of the most important conditions for substituting bone structure by surgical intervention [8]. The biocompatibility of implants must be similar to human bones in terms of the mechanical, chemical, and biological characteristics [9]. Biocompatible materials must perform as a substrate to support the appropriate cellular activity and to optimize tissue generation [10].

The most important polymers that are used in orthopedic implantology are: polymethylmethacrylate (PMMA), silicone rubber, polyethylene (PE), and polyamide (PA) [11,12,13,14]. They are used mainly as “inserts” as the acetabular liner for the orthopedic hip replacement. They also have the advantage of being biocompatible, having a low specific weight, and having a high tensile strength [15]. In implantology, it is very important for every component to ensure the healing process after the surgical operation. The biocompatibility of the implant can be improved by using a mixture of two or more biomaterials in the manufacturing process and having a structural composition that is similar to that of human bone (e.g., hydroxyapatite (HA)) [16,17,18].

There is also the possibility to design different types of patterns on the replacement surface with a good effect on the osseointegration process, and it is also possible to use different manufacturing strategies that do not have a negative effect on the surface quality and the accuracy of the manufactured implant [19]. Polyamide 12 (PA 2200) is a polymer that is often used to produce implants because of its high level of biocompatibility and its mechanical properties. This type of polyamide is suitable for use in the SLS manufacturing process, in all EOSINT P systems with the fine polyamide option, or in all DTM Sinterstation machines [20,21]. Many researchers have tried to find out which is the best material composition, the optimum technological parameters, or the right pattern to apply onto the implant surfaces in order to increase their biocompatibility and mechanical properties [22,23,24].

The mechanical characteristics of the hip joint prostheses made using SLS highly depend on the process parameters, part positioning, and part orientation during the manufacturing process. The main technological parameters of the SLS process are the following: laser power, scanning speed, layer thickness, etc., which should be set up according to the biocompatible powder chosen for the fabrication process [25]. During the last few years, several studies focused on how to determine the proper SLS process parameters for each material used to produce parts using this technology. To determine the influence of the process parameters (laser power, scanning speed) on the mechanical properties and the density and accuracy of the SLS parts, researchers have tried different approaches to obtain better results regarding the process [26,27]. Another approach is to determine the influence of the different positions and orientations of the parts on the building platform. Based on statistical analyses, regression equations were established in which a complex correlation between the process parameters and the part properties was obtained [28,29]. The results of the regression equations showed that for all models, the aspect with highest influence on the target size and mechanical properties was either the scan spacing and the layer thickness or the scan speed. The aspect with the lowest influence was the laser power and the interaction between the powder bed temperature and the layer thickness, while the aspect with the highest influence was attributed to the laser power [30].

The orientation of the parts during the manufacturing process has a high influence on the mechanical strength characteristics. In the case of the z-orientation of the parts, by lowering the temperature, a lower increase of the tensile strength can be observed. Furthermore, for higher temperatures, the flexural strength and compression loading of samples built along the z-axis are usually slightly lower compared to samples built along the x-axis [31,32,33]. The mechanical characterization of medical implants manufactured by SLS can be performed in several ways: by the resonance frequency damping analysis, by standard failure tests, by analytical and numerical methods, or by finite element analysis (FEA). The last method has the advantage that it does not damage the part during the analysis [34,35,36,37]. In biomedical engineering, which involves a combination of engineering and medicine, these physical biomodels, mostly derived from the bone structure, are utilized for the mechanical testing and validation of the computational results obtained during the analysis. The investigation of the biomechanical behavior of an orthopedic medical implant is more complex compared to the mechanical characterization when following standard methods. The biomechanical behavior of a medical implant can be determined by mechanical tests using special devices, by several studies in vivo and in vitro, or by using predictable methods that do not require special conditions, such as FEA methods [38,39,40,41,42,43,44,45]. FEA is one of the most widely used nondestructive methods for the prediction of the mechanical behavior of complex parts or assemblies with relative motions. While functioning, the hip replacement is subjected to several kinds of loads: one is the compressive load, which derives from the contact pressure between the components of the replacement, with a negative impact on the long-term performance [46,47,48].

The research presented in this paper is related to the determination of the optimal parameters and part orientations of implants made of PA 2200 by SLS to achieve the optimal mechanical strength characteristics in terms of compression; the results obtained by the experiments were further used to determine the optimal contact pressure between the components made of PA 2200 by SLS and the components made of TiAl6V4 by SLM and the manufacturing of the acetabular liner by SLS using PA 2200 with the finite element method. Solutions to improve the surface roughness characteristics, as well as the SEM analyses of the samples were considered in the process of manufacturing one medical implant (acetabular liner) made of PA 2200 by SLS using the DTM Sinterstation equipment.

## 2. Materials and Methods

### 2.1. Manufacturing of Samples Made of PA 2200 by SLS and Compression Test Experiments

In the biomedical field, more and more attention has been focused on the resistance of the parts produced by advanced manufacturing technologies. One of the most important characteristics of the parts produced by additive manufacturing technologies that are used as bone replacements is obtaining mechanical properties as close as possible to the characteristics of human bone. In the case of total hip replacement, the plastic liner that is placed between the femoral head and the femoral ball, while functioning, is subjected to compressive loads and contact pressure. Due to this, it is very important to obtain information about the behavior of the material used to manufacture the replacement, under compressive loads. The acetabular liner is usually made of a biocompatible polymer powder such as polymethylmethacrylate (PMMA), silicon rubber, polyethylene (PE), acryl resins, and polyamide (PA). These materials are widely used in additive manufacturing technology, and their mechanical properties are well known. PA 2200 is a new biocompatible, whitish fine powder with high strength and stiffness. This material is biocompatible and presents good mechanical characteristics, closer to other materials used for industrial applications, which makes this powder suitable for medical applications, to produce different type of prostheses and different kinds of inserts between metallic components, as the acetabular liner for example, because of its high abrasion resistance. The mechanical properties given by the producer (EOS, GmbH, Germany) of PA 2200 are given in Table 1.

Several studies have focused on how to obtain information about the tensile strength for different case studies (different orientations of the tested samples, different parameters that were varied on the SLS machine, etc.), as shown in Section 1 of the article, but information about the behavior of the material under compressive loads, which is one of the key pieces of information in the total hip replacement field, is limited in the literature. Therefore, to determine the mechanical characteristics of the samples made of PA 2200 powder under compressive loads, 50 specimens were designed according to the Standard Test Method for Compressive Properties of Rigid Plastics, ISO 604. The specimens had the same rectangular shape and dimensions as follows: 12.7 mm × 12.7 mm × 25.4 mm. To identify the parts after the manufacturing process more easily, the parts were numbered from 1 to 50.

**Figure 1:**
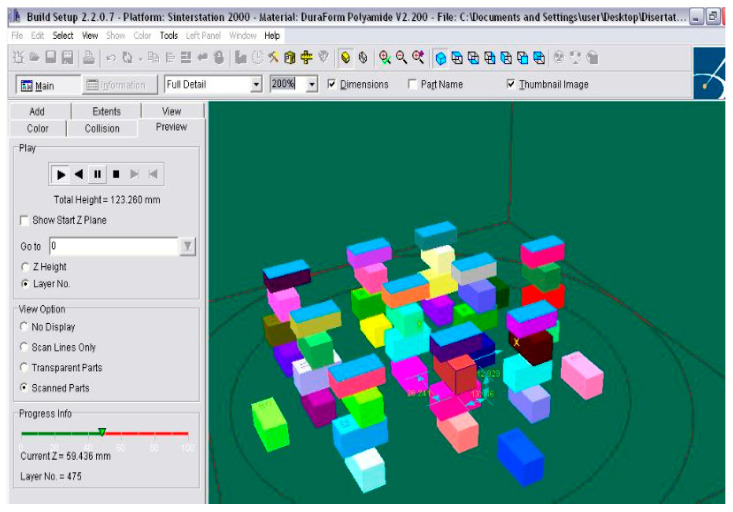
Compressive samples made by the DTM Sinterstation 2000 equipment. Samples’ position on the working area of the machine.

Samples were transferred to the SLS machine, and four types of different orientations of the samples were considered, as shown in Figure 1. The first batch of 10 samples was positioned on the working platform of the machine without any specific orientation, compared to the imported position; only a translation along the z-axis was set as 1 mm from the powder bed.

The second batch of 10 samples was positioned with an orientation of 90° around the y-axis. This batch was placed perpendicular to the first batch. To avoid collision between the batches, the second batch was translated along the z-axis at 20 mm from the powder bed.

The third batch had the same orientation as the first one, but only with a 40 mm translation along the z-axis. The fourth batch was oriented 90° around the y-axis and translated along the z-axis at 50 mm. The fifth batch was realized around the z-axis at −45°, while the sixth batch was oriented around the z-axis at 45°. Different orientations of the samples in the working area of the machine were made based on the idea that the density and mechanical behavior of the laser-sintered parts was nominally a function of different factors, such as the accumulated laser energy density, part bed temperature, and part orientation, according to the literature [46,47,48]. In this sense, the SLS process parameters to build the compressive test samples were established as shown in Table 2. The scanning speed and layer thickness were maintained constant, while the laser power was varied from 4 W to 4.5 W in the case of different batch series. Samples were built using the SLS DTM Sinterstation 2000 equipment, and the result is shown in Figure 1.

**Figure 2:**
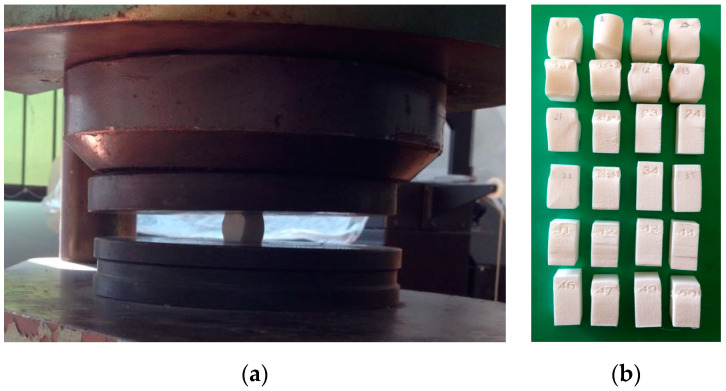
Compressive tests performed in the case of samples made using the DTM Sinterstation 2000 equipment: (**a**) testing equipment on the ZD 40 Tension-Compression Testing Machine; (**b**) 3D-printed samples produced by SLS after the compressive test experiment.

The compression mechanical test was performed by using the ZD 40 Tension-Compression Testing Machine; this type of equipment is suitable for tension–compression testing for different building materials up to a maximum force of 4000 kN. Tests were performed in accordance with the ISO standard test ISO 604 related to Plastics—Determination of compressive properties. The calibration of the machine was performed in accordance with the 50-C0050/CAL procedure for this type of machine [50]. All samples were compressed along the major axis at a constant rate with the displacement equal to 5 mm/min, as shown in Figure 2.

The loads sustained by the samples were measured during the procedure. The universal testing machine stopped applying the load if a failure occurred in the part. According to the standard ASTM D695, in the case of a sample that fails in compression due to a shattering fracture, the compressive strength has a very definite value. In the case of a sample that does not fail in compression due to a shattering fracture, the compressive strength is arbitrary, depending on the degree of distortion, which is regarded as indicating the complete failure of the material.

### 2.2. Microstructural Analyses

For the microstructural study of the PA 2200 compression test specimens made using the SLS technology, a QUANTA FEG 250 SEM microscope was used to investigate the fracture surface due to the compression test. From each batch of samples manufactured with different SLS parameters, only the one that presented the most prominent fracture was chosen for the microscope analyses.

### 2.3. Surface Roughness Evaluation

Besides the mechanical characteristics of the manufactured implants, surface roughness plays one important key role, especially in the case when the surfaces of the realized parts come in contact with other surfaces and are subjected to pressures. This is one of the main reasons why an evaluation of the surface roughness needed to be performed in this case, and solutions for smoothing the surfaces of parts made of PA 2200 also need to be obtained. Since, from the mechanical point of view, the best characteristics were reached in the case of samples realized using a laser power of 4.5 W, the batch of 5 samples manufactured with this setting was considered for this analysis. For each of these samples, Side 1 was considered as being the surface area in direct contact with the powder bed on the working cylinder of the machine (bottom surface), while Side 2 was considered to represent the upper surface of the sample (the last sintered layer of the part). The analysis of the surface roughness was performed using a Mitutoyo SJ 210 Surftest, and for each, 5 points on the two surfaces of each sample and a supplementary analysis of these surfaces (bottom and upper surfaces) were made using the QUANTA FEG 250 SEM microscope. After these analyses, the samples were immersed in a solution of paraxylene solvent for 12 h to improve the surface quality. Surface roughness and SEM analyses were repeated in the same manner using the same methodology. The samples were evaluated before and after the immersion.

### 2.4. Finite Element Method for Optimizing the Contact Pressure between the Acetabular Cup and the Acetabular Liner

**Figure 3:**
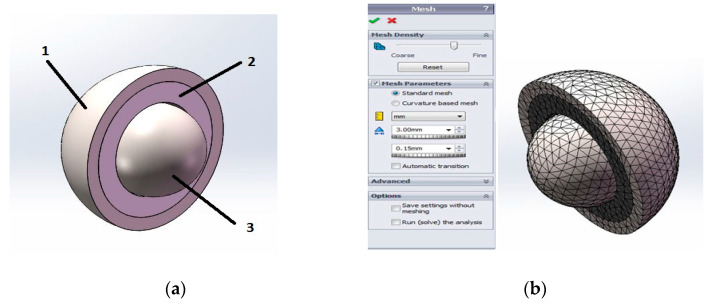
FEA model of the hip replacement: (**a**) hip components: 1—acetabular cup (TiAl6V4); 2—acetabular liner (PA 2200); 3—femoral head (TiAl6V4); (**b**) mesh of the FEA model.

A static study was performed using the SOLIDWORKS Simulation program to estimate the mechanical behavior of the implant made of PA 2200. After the parameters of the study were defined, the material characteristics were specified with linear elastic isotropic specifications, as follows: for both the acetabular cup and the femoral head, a biocompatible titanium alloy was chosen from the SOLIDWORKS Simulation library (Ti6Al4V). Because the material of the plastic liner was not found in the SOLIDWORKS Material Library, a new material definition was necessary for the PA 2200 with its mechanical properties. In order to find a correlation between the results regarding the properties of the PA 2200 samples, the values obtained for the compression test were used for the finite element analysis. The main purpose of the finite element analysis was to analyze the contact pressure between the acetabular cup and the acetabular liner (shown in Figure 3a) in the case of total hip replacements. The next step in the FE analyses was to define the boundary conditions. First, the component contact type was set up to be a global contact with no penetration between the femoral head and the acetabular liner and cup subassembly. A friction coefficient equal to 0.6 µm was chosen according to other research regarding contact pressures between hip replacements components [51]. From the design stage, a boundary condition was given to the acetabular cup and liner assembly, allowing a rotation of 20 degrees around the x-axis (anteversion of the cup) and 45 degrees around the y-axis (cup abduction). To keep the part fixed at the position established as defined in the 3D modeling, the main boundary condition was given as the fixture of the acetabular cup at the end. The external load was applied at a displacement of 0.1 mm in the case of the femoral head. The admissible displacement between the acetabular cup and the femoral head was between 0.03 and 0.35 mm, as specified in the literature [51].

The next step in the FEA model was the meshing procedure, shown in Figure 3b. The mesh parameters were set to 3 mm tetrahedral elements. The tetrahedron is a useful element type for filling in regions with a very complex geometry without sacrificing the element quality. This element type was selected because it can be automatically generated in the software package used for the finite element analysis. This element was selected because it provides good mesh refinements and convergence in this case [52]. After following the steps above, SOLIDWORKS Simulation started to solve the equations and presented the result as displacement, stress, strain, and contact pressure plots. For the calculation of the contact pressure between two spherical components (using the ball and ball–socket model), the numerical calculation of the result was solved by SOLIDWORKS Simulation using Hertzian contact theory.

The curvature sum was calculated using the following formula:

(1) $$\sum p=p_{11}+p_{12}+p_{21}+p_{22},$$

The curvature difference is:

(2) $$F\left(p\right)=\frac{\left(p_{11}-p_{12}\right)+\left(p_{21}-p_{22}\right)}{\sum p},$$

The semimajor and semiminor axes were determined as follows:

(3) $$a=a\sqrt[{*}]{\frac{3Q}{2\sum p}\left(\frac{1-\xi_{1}^{2}}{E_{1}}+\frac{1-\xi_{2}^{2}}{E_{2}}\right)},$$

(4) $$b=b\sqrt[{*}]{\frac{3Q}{2\sum p}\left(\frac{1-\xi_{1}^{2}}{E_{1}}+\frac{1-\xi_{2}^{2}}{E_{2}}\right)},$$

in which: ∑*p* is the curvature sum, *F*(*p*) is the curvature difference, a and b are the semimajor and semiminor axes of the projected contact ellipse, *Q* is the normal force between ball and ball–socket, *E*_1_ and *E*_2_ are the elastic modulus of ball and ball–socket, and *ξ*_1_ and *ξ*_2_ are the Poisson’s ratios of two bodies.

The maximum contact stress between elements was calculated using the following formula:

(5) $$\sigma =\frac{3Q}{2\pi ab}$$

## 3. Results and Discussions

### 3.1. Compression Test Experiments

**Figure 4:**
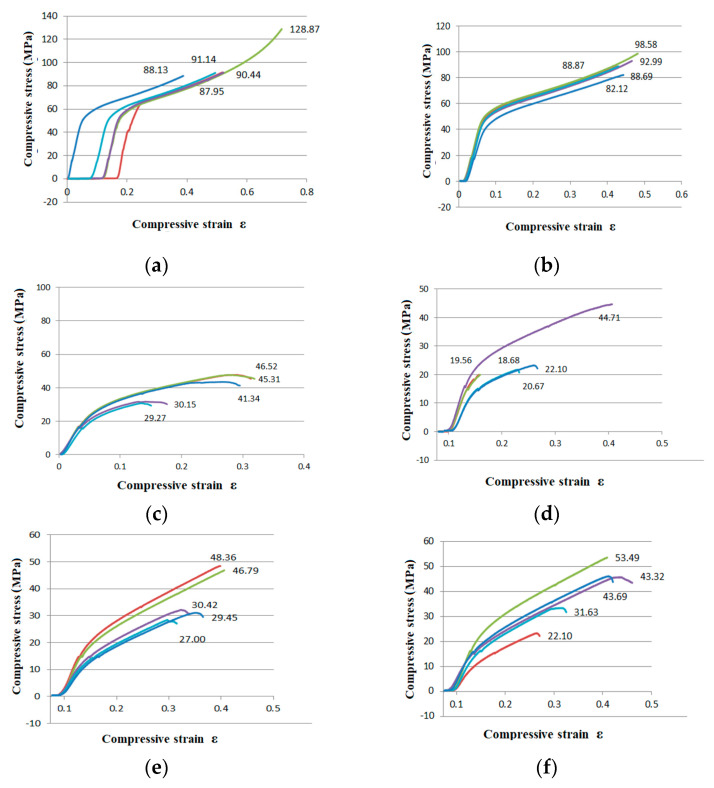
Results of the compressive test experiments: (**a**) first batch series; (**b**) second batch series; (**c**) third batch series; (**d**) fourth batch series; (**e**) fifth batch series (oriented around the z-axis at −45°; (**f**) sixth batch series (oriented around the z-axis at 45°).

The compressive curves of the PA 2200 specimens tested by the compression test are shown for each batch in Figure 4.

The obtained curves showed the typical behavior in compression with few differences regarding the characteristics, as one may notice in Figure 4. The characteristics were determined for samples made of PA 2200 (EOS GmbH, Germany), but built using the SLS DTM Sinterstation 2000 equipment (DTM Corporation, Austin, TX, USA). This demonstrates the importance of the part orientation and technological parameters used to realize the parts by SLS. Five samples of each batch series were tested in order to also check the repeatability of the results. What was really interesting was the fact that using the same technological parameters, but different orientations and positioning of the samples on the working platform of the machine led to different results in terms of the mechanical characteristics, according to the tests that were performed for compression.

As one may notice in Figure 4 that the highest values of the mechanical resistance (before failure) were reached in the case of samples that were made with a P = 4.5 W laser power. The most resistant sample was Sample No. 2, which achieved an R_m_ = 128.87 MPA mechanical resistance (Figure 4a). The lowest value of the mechanical resistance was reached in the case of samples that were made using a P = 4 W laser power and with an orientation of 90° around the y-axis (Sample No. 34; Figure 4d). The value obtained was approximately six-times lower compared to the most resistant sample, R_m_ = 21.78 MPa.

If we consider as a criterion the laser power value used during the manufacturing process, the parts obtained using a P = 4.5 W laser power had the highest resistance compared to the parts obtained using a P = 4 W laser power (see Figure 4c,d). It can be concluded that a laser power of P = 4.5 W is recommended for manufacturing parts from PA 2200 powder by selective laser sintering to reach parts with higher resistance in the end.

If we consider as a criterion the orientation of the parts during the manufacturing process, one may notice that the parts with the standard orientation inside the building chamber (y = 0°) had better mechanical resistance compared to those manufactured with a 90° orientation around the y-axis (see Figure 4a,b). In the case of the orientated part around the z-axis with a 45° inclination, these parts (Figure 4f) had better mechanical resistance compared to those manufactured oriented around the z-axis at −45° (Figure 4e).

### 3.2. Microstructural Analysis

**Figure 5:**
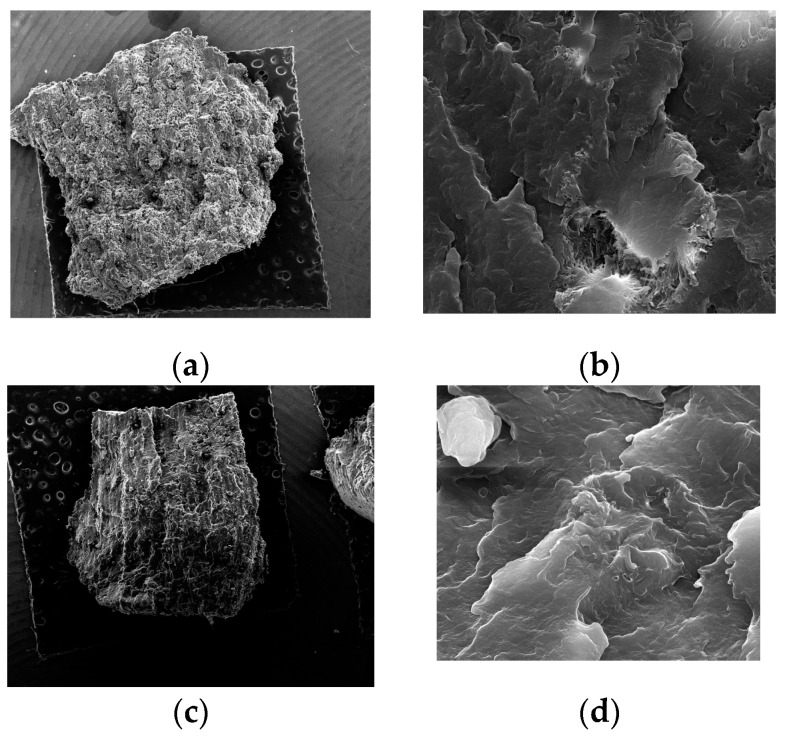

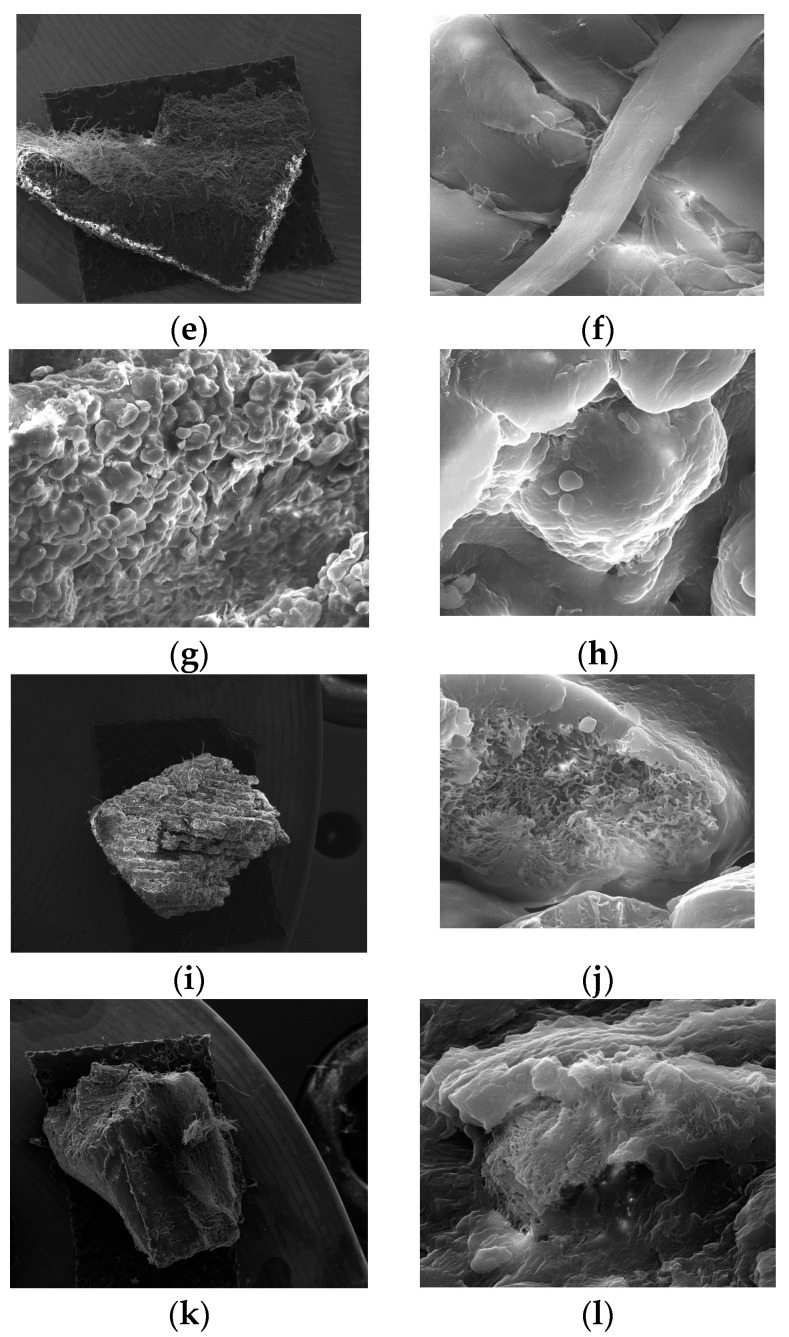
Micrographs of samples made of PA 2200 by SLS: (**a**,**b**) Sample No. 3 made using a P = 4.5 W laser power and y = 0° orientation; (**c**,**d**) Sample No. 15 made by SLS using a P = 4.5 W laser power and y = 90° orientation; (**e**,**f**) Sample No. 25 made by SLS using a P = 4 W laser power and y = 0° orientation; (**g**,**h**) Sample No. 31 made by SLS using a P = 4 W laser power and y = 90° orientation; (**i**,**j**) Sample No. 45 made by SLS using a P = 4.5 W laser power and z = −45° orientation; (**k**,**l**) Sample No. 45 made by SLS using a P = 4.5 W laser power and z = +45° orientation.

The PA 2200 specimens manufactured by the SLS technology were observed under the QUANTA FEG 250 scanning electron microscope (SEM) to investigate the fracture surface resulting from the compression test. In Figure 5, we show the fracture surfaces related to the manufacturing parameters.

From each batch of samples manufactured with different SLS parameters, only the one that presented the most prominent fracture was chosen for the microscope analyses. From the first batch (Samples 1–5) that were manufactured by SLS with a 4.5 W laser power and orientation y = 0°, Sample No. 3 presented the most prominent fracture, which is presented in Figure 5a. A characteristic of laser-sintered components is the presence of pores. Porosity, which is an inherent part of the laser sintering process, has a negative influence on the mechanical properties of the components produced. As can be seen in Figure 5b, increasing the magnitude (5000×), the sintered surface became smoother, and the particles were well united by an intense neck formation. It is possible in this case to notice that the biggest pore dimension was 10 µm, and the distribution was uniform on the fracture surface area.

From the second batch of five samples that were manufactured from PA 2200 powder by SLS, using a P = 4.5 W laser power, and oriented at y = 90°, Sample No. 15 presented the most prominent fracture surface resulting from the compression test. The fracture surface of Sample 15 is presented in Figure 5c. As can be seen in Figure 5c, the distribution of the pores was uniform on the fracture surface. Increasing the magnification (5000×) of the SEM up to 30 µm (Figure 5d), the building of the layer could be observed. The interconnected pores distributed throughout the fracture surface can be identified. It can be seen that the powder particles were fused together within one layer, and no secondary pores can be observed on the sintered layer, which may affect the mechanical resistance.

From the third batch of five samples made from PA 2200 by the selective laser sintering technology, using a P = 4W laser power and oriented at y = 0°, Sample No. 25 presented the most prominent fracture surface resulting from the compression test, as presented in Figure 5e. Increasing the magnitude (5000×) (Figure 5f), four individual layers without detached pores on the fracture surface can be identified. It can be seen that the powder particles were fused together within one layer, but the connection with the adjacent layers was not well defined. The presence of the individual layers and relatively large gaps between them could result from the insufficient laser power used during the sintering process, which reduced the strength of the part and had a negative influence on the mechanical properties.

From the fourth batch of five samples made from PA 2200 by the selective laser sintering technology, using a P = 4W laser power and oriented at y = 90°, Sample No. 31 presented the most prominent fracture surface resulting from the compression test, as shown in Figure 5g. As can be seen in Figure 5h, the sintered layer’s surface was smooth without partially sintered pores that could adhere to the surface. The presence of pronounced voids between two layer can be identified, which was due to the low-power beam used in the manufacturing stage or possibly due to the severe cooling of the material. It can be seen that only a low fraction of the particles liquefied; therefore, their spherical shape can still be easily seen. The powder particles were loosely bound, and the majority of the particles can be recognized individually due to the decrease of the laser power. The presence of these pores, which formed a bridge between sintered pores at 10–30 µm, was visible in the material structure, while the grains were detached, forming secondary pores, forming a pronounced porous surface with a negative influence on the mechanical resistance.

From the fifth batch of five samples made from PA 2200 by the selective laser sintering technology, using a P = 4.5 W laser power and oriented at z = −45°, Sample No. 45 presented the most prominent fracture surface resulting from the compression test, which is shown in Figure 5i. In Figure 5i, we show the fracture zone of the sample resulting from the compression test. The orientation of the individual layers can be identified clearly, which were oriented at −45° around the z-axis of the machine during the manufacturing process. Because of the orientation of the part during the manufacturing process, large gaps can be identified between the layers. Increasing the magnitude (5000×), as shown in Figure 5j, a pronounced superficial porous surface can be identified. The grains adhered to the sintered surface, forming bridges with the adjacent pores with a size between 5 and 20 µm, which could cause further detachment of the particles, affecting the mechanical resistance. Some of the particles could be embedded in the “parent particle”. The size of the contact formed between two adjacent cells was between 5 and 10 µm.

From the sixth batch of five samples made from PA 2200 by the selective laser sintering technology, using a P = 4.5 W laser power and oriented at z = +45°, Sample 46 presented the most prominent fracture surface resulting from the compression test, as shown in Figure 5k. In this figure, the quality of the surface obtained by the selective laser sintering technology can be seen. The grains were fused together, forming a strong connection between two adjacent layers. The presence of the secondary pores (Figure 5l) that formed the bridge connection with the sintered pores had no significant influence on the mechanical properties of the parts. The average pore size was between 10 and 15 µm.

By the SEM analyses of the samples manufactured using different processing parameters and orientations of the samples in the workspace of the SLS machine, it was concluded that the parts with a laser power P = 4.5 W had a smoother surface, fewer pores, and a smaller distance between the layers compared to those manufactured with a P = 4 W laser power. The orientation of the samples had a significant influence on the connection between two adjacent sintered layers. In the case of the orientation at y= 0°, perpendicular to the scanning direction, the layers were easily identified individual. The fusion between the layers for the parts oriented at y = 90° was more pronounced, without significant gaps between two adjacent layers. In the case of the parts oriented at z = +45° and z = −45°, the distance between two adjacent layers increased, and a pronounced stair effect appeared, which had a negative influence on the mechanical resistance of the parts. Comparing all the samples analyzed with the QUANTA FEG 250 SEM, it can be concluded that Sample No. 15 from the second batch had the most uniform pore distribution, while the fusion of the layer was more pronounced, and no secondary pores with a negative influence on the mechanical strength were observed in this case.

### 3.3. Surface Roughness of PA 2200 by SLS

In the literature, there are studies on the use of parylene as the final coating, in order to protect the devices, components, and surfaces in the medical and engineering industry [53,54]. Therefore, an attempt was made to treat the surface manufactured from PA 2200 at 4.5 W with paraxylene. The samples were immersed in the paraxylene solution for 12 h at room temperature. Samples were measured before and after immersion on each side (bottom and upper surface), and the results obtained (calculated by averaging, taking into consideration both sides) are presented in Table 3.

To compare the results, a statistical analysis was performed. Two hypotheses were considered: (H0) there is no significant difference in the roughness between the control group and the paraxylene-treated group; and (H1) there is a significant difference in roughness between the control group and the paraxylene-treated group. To test these hypotheses, a two-tailed independent sample t-test was conducted with a significance level of α = 0.05. The average roughness was different in the control group for Side 1 (M = 13.7 µm, SD = 0.361) and Side 2 (M = 11.504 µm, SD = 0.350). In the case of Side 1, there was a significant difference in the values of the roughness Ra of Control Group 1 (M = 13.7 µm, SD = 0.361) and the roughness of the surface treated with paraxylene (M = 12.387, SD = 0.46); t(4) = 8.19, p = 0.0012). In the case of Side 2, there was also a significant difference between the values of the roughness Ra of Control Group 1 (M = 11.504 µm, SD = 0.350) and the surface treated with paraxylene (M = 10.831, SD = 0.22); t(4) = 40.46, p = 0.00000022). The results obtained showed that H1 was true, meaning that the paraxylene solution affected the surface of the samples, changing the Ra values for both sides of the samples. As can be observed, the paraxylene substance decreased the Ra values, meaning that the surface would be smoother. This can represent an advantage for the coated material obtained taking into account the medical field, where the future implant will be used. The research in this field showed that the adhesion of the cells is also influenced by the Ra values of the implant surface [55,56]. The surface roughness of the future implant must be adapted to the cell dimensions (of the neighboring tissue of the body) for better adhesion. By comparing these results to the ones obtained by other researchers in this domain [57], it was noticed that the surface quality that was reached in this case was improved compared to those of parts produced using PA 2200 with other types of SLS equipment. The differences came from the fact that the type of equipment and the performance of the equipment (Formiga P110 from EOS and DTM Sinterstation 2000) were slightly different, even if the material powder (raw powder PA 2200) was the same in these cases that were compared.

**Figure 6:**
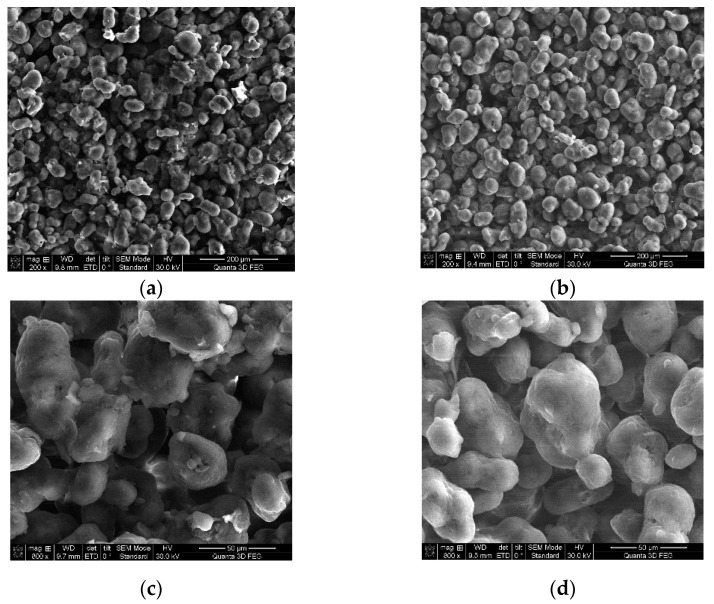
SEM analysis before and after immersion in the paraxylene solution: (**a**) before immersion (200×); (**b**) after immersion (200×); (**c**) before immersion (800×); (**d**) after immersion (800×).

To investigate the effect of paraxylene on the surface roughness of the samples obtained by the SLS technology using PA 2200, we used the SEM analysis on the microstructure of the samples. In Figure 6, it is possible to notice that the particle surface was cleaner after immersion than before, a fact that increased the surface quality of the implants.

### 3.4. Finite Element Analysis of the Orthopedic Implant Made from PA 2200 by SLS

**Figure 7:**
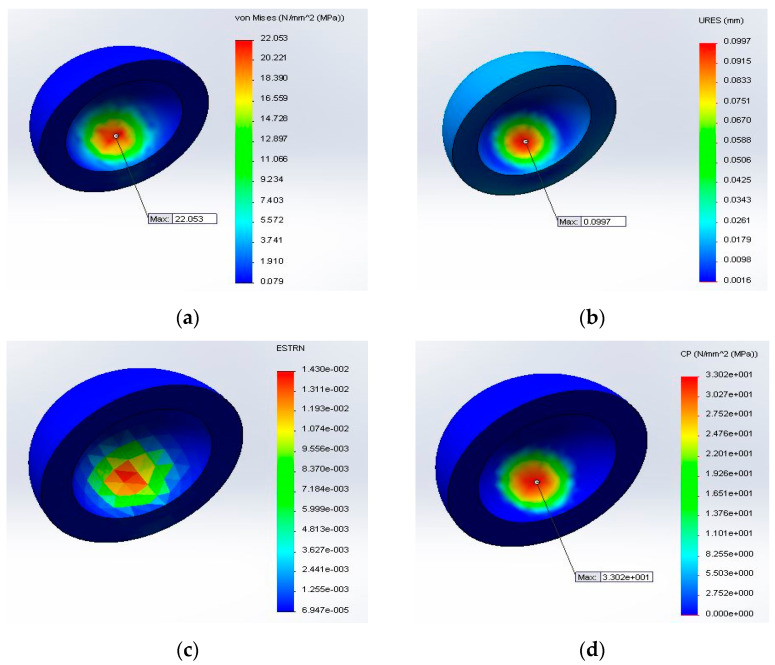
FEA results: (**a**) distribution of the von Mises stress on the PA 2200 acetabular liner; (**b**) distribution of the displacement; (**c**) distribution of the deformation; (**d**) distribution of the contact pressure on the acetabular liner’s inner surface.

The FE analysis was performed using the characteristics obtained in the mechanical compression tests. The value of the displacement applied as the external load was equal for each test: 0.1 mm as the advanced boundary condition, the maximum value obtained being 0.0977 mm, as shown in Figure 7b.

To determine the contact pressure of the PA 2200 acetabular liner, a finite element analysis using SOLIDWORKS Simulation was performed, the elastic Young’s modulus determined by the compression test being transposed in the FEA material definition to determine the correlation between the mechanical properties obtained from the PA 2200 samples and the behavior of the acetabular cup. The contact pressure that was reached using the characteristics determined in the case of samples made with P = 4.5 W was within the admissible limits, while for the samples manufactured with a laser power equal to 4 W under the same loading conditions, it was concluded that these parts would not resist high compression values. Taking into consideration all these aspects, the acetabular liner was manufactured from PA 2200 by SLS on the DTM Sinterstation 2000 equipment using the processing parameters of laser P = 4.5 W and an orientation of y = 90° inside the building chamber of the machine, the acetabular cup being immersed in the paraxylene solution for 12 h at room temperature, as for the analyzed samples.

The distribution of the deformations obtained by the FEA analysis were the same for each sample (Figure 7c), the maximum value obtained being 1.430 × 10^−2^ mm.

The maximum value of the von Mises stress was obtained for Sample 1 from the first batch of samples manufactured by SLS, which is shown in Figure 7a (*σ_max_* = 22.053 MPa). The results of the finite element analysis are shown in Table 4, in which is presented the maximum von Mises stress (*σ_max_*) and contact pressure (*C_p_*) for each batch.

One of the most important conditions for a part to resist pressure is to satisfy the following condition:

(6) $$\sigma_{max}\leq \sigma_{a},$$

(7) $$\sigma_{a}=\frac{Rdx}{c},$$

in which: *σ_max_* is the maximum von Mises stress obtained by the FEA [MPa], *σ_a_* the admissible stress (MPa), *Rdx* the compression limit strength (MPa), and *c* the safety coefficient (*c* = 1.5).

As one may notice in Table 4 from the mechanical characteristics’ point of view, only the first two batched of PA 2200 used for the orthopedic implant FEA analysis respected the condition explained by Equation (6) (22.53 MPa ≤ 24.22 MPa and 18.33 MPa ≤ 21.97 MPa).

For the samples manufactured with a laser power equal to 4 W under the same loading condition, Relation (6) was not respected; therefore, it can be concluded that these parts would not resist high compression.

For the mechanical characteristics obtained for the batch manufactured with a rotation around the z-axis of 45°, the values of the maximum von Mises stress were very close to the admissible stress. After comparing the results obtained by the finite element analysis, it can be concluded that the SLS process parameters used for the first two batches had the proper mechanical behavior, suitable to use to manufacture orthopedic acetabular cups. The contact pressure is directly proportional to the elastic Young’s modulus according to Hertzian Equation (4). The effects of the design parameters, such as the radial clearance, the bone quality, the coefficient of friction, and the cup–bone interface, on the contact mechanics at the bearing surfaces could be important for the clinical performance and long-term survival of the prosthesis [52]. The results of the contact pressure (Figure 7d) can be compared to those obtained by other researchers that performed a comparison between two polyethylene acetabular cups with different dimensions (36 mm and 40 mm), for which they obtained a range of contact pressure between 8.2 and 60.4 MPa [7,58,59,60,61,62].

Considering the mechanical properties determined by the compression test performed on the universal testing machine for each batch of five samples manufactured by the selective laser sintering technology using different process parameters, after comparing the results obtained by the finite element analysis of the acetabular liner performed with the SOLIDWORKS Simulation CAE software for each mechanical property determined by the compression test, as well as after the results obtained by the mechanical characterization of the PA 2200 biocompatible powder performed by the QUANTA 3D FEG SEM microscope, the result was that the most resistant components manufactured from PA 2200 by SLS were those obtained with a laser power P = 4.5 W and with an orientation around the y-axis (y = 90°) inside the building chamber. Considering the results obtained and presented in the previous sections, the customized orthopedic implant was manufactured from the PA 2200 biocompatible material by selective laser sintering technology on the DTM Sinterstation 2000 using the technological parameters shown in Table 5.

**Figure 8:**
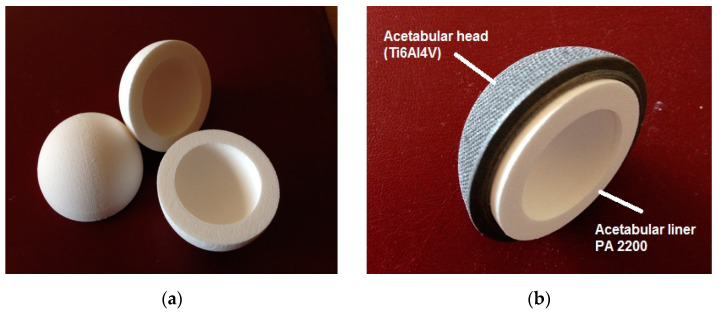
(**a**) Customized medical implant made of PA 2200 by the SLS technology; (**b**) acetabular liner made by the SLS technology and acetabular head made of TiAl6V4 material by SLM 250 HL from SLM Solutions.

The acetabular liner designed for a customized orthopedic hip replacement made by SLM technology from Ti6Al4V was successfully manufactured by SLS from the PA 2200 biocompatible material on the DTM Sinterstation 2000 equipment as shown in Figure 8a. The acetabular head shown in Figure 8b was designed and realized for a customized hip replacement, the acetabular head cup being made from Ti6Al4V powder using the selective laser melting technology.

## 4. Conclusions

After performing the FEM analysis and mechanical tests, we can conclude that the strongest samples in compression were the ones obtained with a P = 4.5 W laser power and an orientation y = 90° (R_m_ = 128.87 MPa). The lowest value of the mechanical resistance was obtained in the case of the fourth batch of samples that was manufactured with a P = 4 W laser power and with an orientation of 90° around the y-axis. The value obtained was approximately six-times lower compared to the most resistant sample, R_m_ = 21.78 MPa. The microstructural properties of the fracture zone performed using SEM revealed that the most uniform distribution of the pores and the fusion between adjacent layers were reached in the case of samples made with a P = 4.5 W laser power and orientation y = 90°.

The immersion in the paraxylene solution for 12 h at room temperature had one benefit concerning the surface roughness. The measuring tests that were performed on the upper and bottom layers of the samples, followed by the statistical analysis of the roughness (Ra) showed there was a significant difference between the roughness of the control group surface (untreated) and the roughness of the samples treated with the paraxylene solution for both sides of the samples, emphasizing that the paraxylene solution can influence the roughness of the future implant and can be an adequate solution for the cases when a smooth surface is needed. Using SEM analysis, we noticed that the particles surface was cleaner, which could lead to a better surface quality of the implants.

Future investigations will be addressed to testing the benefits and influence of using paraxylene and other similar materials on the biocompatibility and bioactivity of the realized implants by SLS. Furthermore, different coating methods applied on the surfaces of the implants realized by SLS for the same purposes are worth future investigation.

## Figures and Tables

**Table 1:** Mechanical and thermal characteristics of PA 2200 [49].

Mechanical Properties		Value	Unit	Test Standard
Flexural modulus, 23 °C		1500	MPa	ISO 178
Flexural strength		58	MPa	ISO 178
Izod impact notched, 23 °C		4.4	kJ/m^2^	ISO 180/1A
Izod impact unnotched, 23 °C		32.8	kJ/m^2^	ISO 180/1U
Shore D hardness (15 s)		75	-	ISO 868
Ball indentation hardness		78	MPa	ISO 2039-1
**3D Data**		**Value**	**Unit**	**Test Standard**
Tensile modulus	X-direction	1700	MPa	ISO 527-1/-2
	Y-direction	1700	MPa	ISO 527-1/-2
	Z-direction	1650	MPa	ISO 527-1/-2
Tensile strength	X-direction	48	MPa	ISO 527-1/-2
	Y-direction	48	MPa	ISO 527-1/-2
	Z-direction	47	MPa	ISO 527-1/-2
Strain at break (X-direction)		24	%	ISO 527-1/-2
Charpy impact strength (+23 °C, X-direction)		53	kJ/m^2^	ISO 179/1eU
Charpy notched impact strength (+23 °C, X-direction)		4.8	kJ/m^2^	ISO 179/1eA
Thermal conductivity	X-direction	0.144	W/(mK)	DIN 52616
	Y-direction	0.144	W/(mK)	DIN 52616
	Z-direction	0.127	W/(mK)	DIN 52616
**Thermal Properties**		**Value**	**Unit**	**Test Standard**
Melting temperature (10 °C/min)		176	°C	ISO 11357-1/-3
Vicat softening temperature A		181	°C	ISO 306
Vicat softening temperature (50 °C/h 50 N)		163	°C	ISO 306

**Table 2:** SLS process parameters of Compressive test samples.

Sample No.	Laser Power (W)	Scan Speed (mm/s)	Layer Thickness (mm)	Orientation
Samples 1 to 10	4.5	1257.3	0.1	y = 0°
Samples 11 to 20	4.5	1257.3	0.1	y = 90°
Samples 21 to 30	4	1257.3	0.1	y = 0°
Samples 31 to 40	4	1257.3	0.1	y = 90°
Samples 41 to 45	4.5	1257.3	0.1	z = −45°
Samples 46 to 50	4.5	1257.3	0.1	z = +45°

**Table 3:** Roughness values (Ra) for the PA 2200 samples manufactured at 4.5 W.

	Side 1		Side 2	
	Control Group	Paraxylene	Control Group	Paraxylene
Sample No.	Average Value	Average Value	Average Value	Average Value
1	13.756	12.456	11.510	10.789
2	13.473	11.897	11.921	11.135
3	13.210	11.958	10.961	10.546
4	13.960	12.650	11.478	10.748
5	14.100	12.976	11.652	10.936
Mean	13.700	12.387	11.504	10.831
Standard deviation	0.361	0.460	0.350	0.22
*p*-value (<0.05)		0.001209295		2.22946 × 10^−6^
t-value		8.192505921		40.46192006

**Table 4:** Mechanical characteristics determined by FEA for the hip implant prostheses.

PA 2200 Samples	*R_dx_* (MPa)	*σ_a_* (MPa)	*σ_max_* (MPa)	*C_p_* (MPa)
I (1–5)	36.3	24.22	22.053	33.02
II (11–15)	32.95	21.97	18.33	27.45
III (21–25)	15.23	10.15	10.60	15.88
IV (31–35)	12.08	8.05	9.24	13.84
V (41–45)	10.63	7.08	6.57	9.83
VI (46–50)	10.81	7.20	7.08	10.59

**Table 5:** Technological parameters used for manufacturing the acetabular liner by SLS.

Laser Power (W)	Scanning Speed (mm/s)	Scanning Distance (mm)	Layer Thickness (mm)	Temperature (°C)	Orientation (°)
4.5	1257.3	0.15	0.1	170	y = 90°

## Data Availability

Not applicable.
