# Correction: Răzvan et al. Selective Laser Sintering of PA 2200 for Hip Implant Applications: Finite Element Analysis, Process Optimization, Morphological and Mechanical Characterization. *Materials* **2021**, *14,* 4240

**DOI:** 10.3390/ma15010132

**Published:** 2021-12-24

**Authors:** Răzvan Păcurar, Petru Berce, Anna Petrilak, Ovidiu Nemeş, Cristina Ştefana Miron Borzan, Marta Harničárová, Ancuţa Păcurar

**Affiliations:** 1 Department of Manufacturing Engineering, Faculty of Industrial Engineering, Robotics, Management and Production Management, Technical University of Cluj-Napoca, B-dul Muncii 103–105, 400641 Cluj-Napoca, Romania; petru.berce@tcm.utcluj.ro (P.B.); anna_petrilak@yahoo.com (A.P.); 2 Department of Environmental Engineering and Sustainable Development Entrepreneurship, Faculty of Materials and Environmental Engineering, Technical University of Cluj-Napoca, B-dul Muncii 103–105, 400641 Cluj-Napoca, Romania; ovidiu.nemes@imadd.utcluj.ro; 3 Department of Electrical Engineering, Automation and Informatics, Faculty of Engineering, Slovak University of Agriculture in Nitra, Tr. A. Hlinku 2, 949 76 Nitra, Slovakia; marta.harnicarova@uniag.sk; 4 Department of Mechanical Engineering, Faculty of Technology, Institute of Technology and Business in České Budějovice, Okružní 10, 370 01 České Budějovice, Czech Republic

The authors wish to make the following correction to their paper [1]:

**Incorrect Title**

There is an error in the title. The correct title of the article is “Selective Laser Sintering of PA 2200 for Hip Implant Applications: Finite Element Analysis, Process Optimization, Morphological and Mechanical Characterization”. We apologize for this error and state that the scientific conclusions are unaffected. The original article has been updated. 
